# Continuous and Periodic Monitoring System of Surface Water Quality of an Impounding Reservoir: Sulejow Reservoir, Poland

**DOI:** 10.3390/ijerph16030301

**Published:** 2019-01-23

**Authors:** Aleksandra Ziemińska-Stolarska, Mirosław Imbierowicz, Marcin Jaskulski, Aleksander Szmidt, Ireneusz Zbiciński

**Affiliations:** 1 Faculty of Process and Environmental Engineering, Lodz University of Technology, 90-924 Lodz, Poland; miroslaw.imbierowicz@p.lodz.pl (M.I.); ireneusz.zbicinski@p.lodz.pl (I.Z.); 2 Faculty of Geographical Sciences, University of Lodz, 90-139 Lodz, Poland; marcin.jaskulski@geo.uni.lodz.pl (M.J.); aleksander.szmidt@geo.uni.lodz.pl (A.S.)

**Keywords:** online monitoring, dam reservoir, water quality, multi-parameter probe, eutrophication

## Abstract

The paper presents results of water quality monitoring conducted within the frame of the MONSUL project. The main goal was to analyse and assess the impact of factors determining the ecological status of a dam reservoir on the basis of the Sulejow Reservoir located in Central Poland. The project implementation plan based on comprehensive research-based monitoring covered the following parameters characterising the ecological potential of the reservoir: water temperature, pH, oxygen concentration, chlorophyll “a” and blue-green algae, concentration of ammonium ion, nitrate nitrogen phosphates as well as total organic carbon, chemical oxygen demand and biochemical oxygen demand. The parameters were measured with a mobile and stationary monitoring system and supplemented by an off-line analysis of water samples in the laboratory. The study was carried out during two seasons: May–October 2015 and April–November 2016; the results were analysed also with regard to the weather conditions. Despite the similar temperatures of water and air in the analysed seasons, significant differences were observed for atmospheric precipitation; 2015 was a dry year, and the climatic water balance for the analysed area was negative, which caused limited surface runoff and decreased the concentrations of nutrient in the reservoir waters. Data from continuous monitoring, supplemented with the results of laboratory measurements, indicated that the values of TOC (Total Organic Carbon) and COD (Chemical Oxygen Demand) parameters were within the purity class I; exceedances refer to the BOD (Biochemical Oxygen Demand) value, which confirmed the presence of biodegradable organic compounds in the reservoir waters. The values of chlorophyll “a” and the presence of algae during the vegetation season testify to eutrophication of the Sulejow Reservoir.

## 1. Introduction

In contrast to flowing waters, lakes, dam reservoirs and impoundments were not emphasized in the early years of water quality modelling, as this issue has never been the major focus of urban development. Starting in the 1970s, however, it was recognized that natural and man-made lakes are equally, if not more important than estuaries and rivers, from a recreational standpoint [1]. Dam reservoirs enable the production of low-cost and environmentally friendly electricity, fish farming, as well as creating new landscape features and enhancing the attractiveness of the adjacent areas. By regulating the flow, most reservoirs serve the purpose of flood control and make it possible to raise the minimum water level in the river, in case of drought. Many dam lakes also play an important economic function, supplying drinking water to large urban agglomerations.

Dam reservoirs represent a unique ecosystem, which was developed by the imposition of natural characteristics of the feeding river and modification of the natural conditions, caused by human interference, e.g., a sedimentation area of mineral, organic matter and pollutants carried by the inflow rivers, which changes the trophic status of the water body. The character and magnitude of changes depend on the characteristic parameters of the reservoir (surface, shape, depth, expansion of the littoral zone, water-level fluctuations), as well as size and quality of the supplying river.

The capacity of artificial reservoirs in Poland corresponds to about 6% of the annual river runoff (compared with 10%–12% in the neighbouring countries) [2]. In Poland more than 100 artificial dammed reservoirs are located, with a total surface area of about 350 km^2^, representing approx. 0.11% of the Polish territory.

A dam reservoir represents a more complex hydrodynamic system in comparison with the lake, characterized by a dynamic inflow of water, with a large amount of debris and contaminants. A continuous supply of nutrients with river waters causes a significant increase in the trophic status of the reservoir waters. Quantitative and qualitative supply of reservoirs with nutrients and other chemical compounds, depend on the size and the way of catchment management, loading of the reservoir with point-and surface run-offs, as well as, on the amount and composition of rainfalls and groundwater, which supply the water body [3,4]. For this reason, the quality of water in artificial water reservoirs should be constantly monitored to undertake specific practical actions to improve water quality in a timely manner.

The monitoring of water quality is very important for maintaining the safety of water resources used for various purposes such as drinking, recreation or fish farming [5,6]. Monitoring long-term trends and changes in water quality is the first step towards a deeper understanding of the sources of pollution and it can lead to smart water management and informed decision making.

In addition, continuous online monitoring solutions can provide early warning process adjustments for incoming algae blooms and turbidity events [7].

The European Union Water Framework Directive (2000/60/EC) has created a demand for water quality monitoring systems to monitor reliably a larger number of water quality parameters at regular intervals. This requires trained personnel, reliable instrumentation and in the long run high laboratory costs to achieve the expected monitoring goals. To achieve the WFD-targets (Water Framework Directive), there is a need for the development of a cost-effective monitoring system using advanced technology to reduce overall analytical costs.

Legal monitoring, carried out on dam reservoirs with a frequency of once a quarter, may not give a full picture of changes in water quality. This refers to indicators of high diurnal variation, such as conductivity, chlorophyll concentration and dissolved oxygen. In contrast to periodic monitoring, continuous monitoring provides results with an incomparably better time resolution. This allows a more accurate representation of hydro chemical phenomena in the reservoir models, and gives the chance to detect momentary and fast-changing phenomena [8]. Automatic monitoring is limited to a small number of indicators. Periodic monitoring can be conducted for a very large number of indicators that determine the dynamics of changes in the long-term horizon (e.g., on a yearly basis).

### Study Area

Sulejow Reservoir is situated in the voivodeship of Lodz (51°22’–51°28’ N, 19°51’–20°01’ S). One of the biggest artificial reservoirs in Poland was built by impounding the Plica River at 138.9 km with a dam, in the years 1969–1973. It is a typical lowland, low volume reservoir (75 milion m^3^) with major fluctuations of water level. As a shallow water body (mean depth 3.3 m), it covers a large area (22 km^2^). Average water retention time, of several years is 40 days, which causes total water exchange in the reservoir to take place nine times a year. The banks of Sulejow Reservoir are regulated at 23 km, which is about 40% of the total shoreline length; the remaining 35 km is forested. The entire catchment of the discussed reservoir has an agricultural and forest character. Cultivated lands covered 64.21% and forests 30.69% of the total basin area. A similar structure is shown by the arable lands in the vicinity of the reservoir (19.9 km^2^), where the fields pose 60.03%, and forests 37.43% respectively. In the Sulejow Reservoir, a ribbon-type, artificial lake, two morphological zones can be distinguished, each influenced by different driving forces. The first one (consisting of a riverine and a transition zone) is the narrow, shallower part of the reservoir, dominated by the river inflows. The second, a wide, lacustrine zone, located near the dam is open and behaves as a lake. The main mechanism causing the mixing of water masses is wind. The main axis of the reservoir runs from southwest to north-east, which is close to the direction of the winds that ripple and mix the water. As a result, formation of stagnant water zones, on the southern bank of the middle and lower part of the reservoir occurs [9,10].

## 2. Method and Scope of Completed Studies

The monitoring program carried out within the MONSUL project consisted of three interconnected research platforms:

a) stationary monitoring system, EXO2 probe (Xylem Inc., Rye Brook, NY, USA) installed on a buoy anchored near the dam,
b) mobile measurement system, based on the EXO2 probe mounted on a motorboat,
c) off-line measurement system, samples of water collected from the reservoir and analysed in a laboratory.

The monitoring was carried out during two study seasons: from May to October 2015 and from April do November 2016.

### 2.1. Research-Based Stationary Monitoring System

The stationary monitoring system, consisted of a probe mounted on a buoy, anchored near the dam, equipped with 7 sensors enabling continuous, automatic measurement of the following parameters: temperature, pH, concentration of dissolved oxygen, conductivity, chlorophyll concentration, algae concentration (blue-green algae, BGA) and concentration of ammonium ion (Table 1). The device includes also a meteorological sensor measuring the following parameters in a continuous mode: water temperature and humidity, wind speed and direction, atmospheric pressure and sunlight.

The measurement probe was immersed in the water to a depth of approx. 3 m. The EXO2 probe and sensors were manufactured by the YSI company (Yellow Springs, OH, USA). Digital smart sensor featuring titanium construction and wet-mateable connectors were connected to the probe. In the implementation of continuous monitoring by means of automatic measuring devices, attention should be paid to strict compliance with sensor calibration procedures, probe cleaning and periodicity, according to the instructions, sensor replacement. The probe was calibrated every two months based on relevant procedures and benchmark solutions supplied by the producer (https://www.ysi.com/EXO2) [11]. A rotating brush installed in the central port of the probe helped to remove fouling from the sensors. The wiper was manually activated from a PC and turned on at an interval of 15 min. In addition to the wiper the probe was cleaned manually, twice during the measurement season due to the intensive algae bloom. Sensor replacement took place after two monitoring seasons according to the supplier’s recommendations.

**Figure 1:**
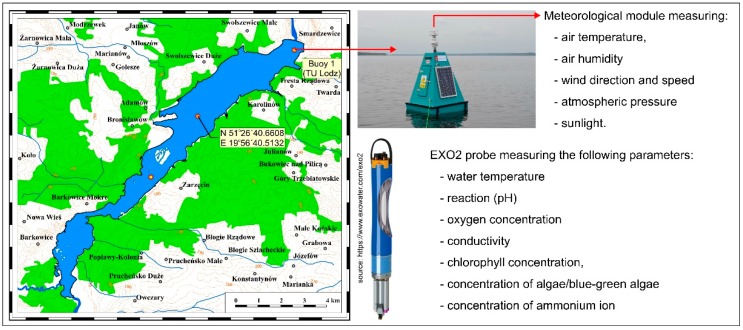
Localization and photography of the stationary system for water quality measurements, installed on the Sulejow Reservoir.

The multi-parameter probe and a data logger wirelessly transmit the data to the hosted data collection platform so that readings can be viewed on the project website: www.monsul.wipos.p.lodz.pl [12]. The location of the stationary measurement system and photo of the EXO2 probe are presented in Figure 1.

**Figure 2:**
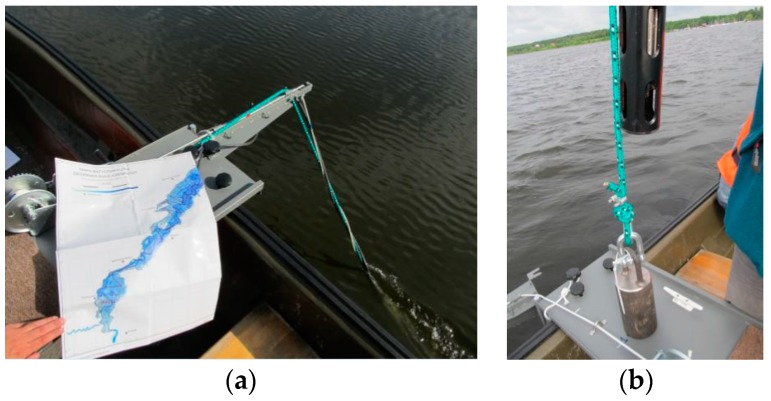
Mobile monitoring measurement system, (**a**): Bench with an extension arm; (**b**): probe with a weight.

The mobile monitoring measurement system consisted of the abovementioned EXO2 probe and GPS module enabling identification of the position of the probe at the time of measurement. A special “bench with an extension arm” was built for the measurements in the flow and installed on a motorboat (Figure 2). The system allowed floating across the lake traversing approx. 17 km and measuring the water quality parameters. The measurement data were collected in the cache of a portable data logger from where they were sent to a PC.

In order to minimize any potential errors, initial tests were performed to identify the most relevant conditions for performing mobile measurements. The purpose of such tests was to evaluate the impact of the velocity of the motorboat and the depth of the probe immersion on the results and reliability of the acquired data. Measurements of the water quality parameters were made with a boat speed of 5.5–6 km/h and 9.0–9.5 km/h. Additional measurements were performed in the same area with a fixed probe immersed in water to the depths of h = 3.5 m and h = 1.5 m. A statistical analysis revealed that if the speed of the boat did not exceed 6 km/h, the results of measurements performed with a motorboat with the quoted speed did not vary from the results of measurements performed with a fixed probe. If the speed of the boat increased to 9 km/h deterioration of measurements accuracy was observed. According to the analysis, a reduction in the immersion depth of the probe from 3.5 m to 1.5 m did not significantly affect the results of measurements [13].

**Figure 3:**
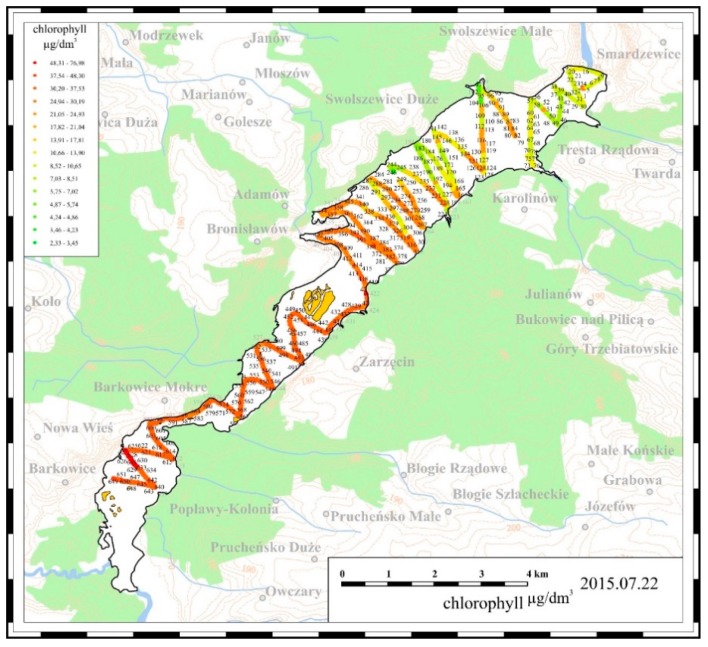
Profile of changes in chlorophyll concentration (μg/dm^3^) in the area of the Sulejow Reservoir, obtained with a mobile measurement system on 19 July 2016.

The mobile measurements delivered information about water quality in 650–700 points located all over the reservoir. An example of the measurement data obtained by the boat floating across the reservoir is presented in Figure 3. The data refer to chlorophyll concentrations measured on 19 July 2016:

Collections of measurement data were stored in the ArcGIS database, and after being processed with relevant algorithms, they were used for visualization of the spatial distribution of the parameters measured along the reservoir.

### 2.2. Laboratory Test Method

**Figure 4:**
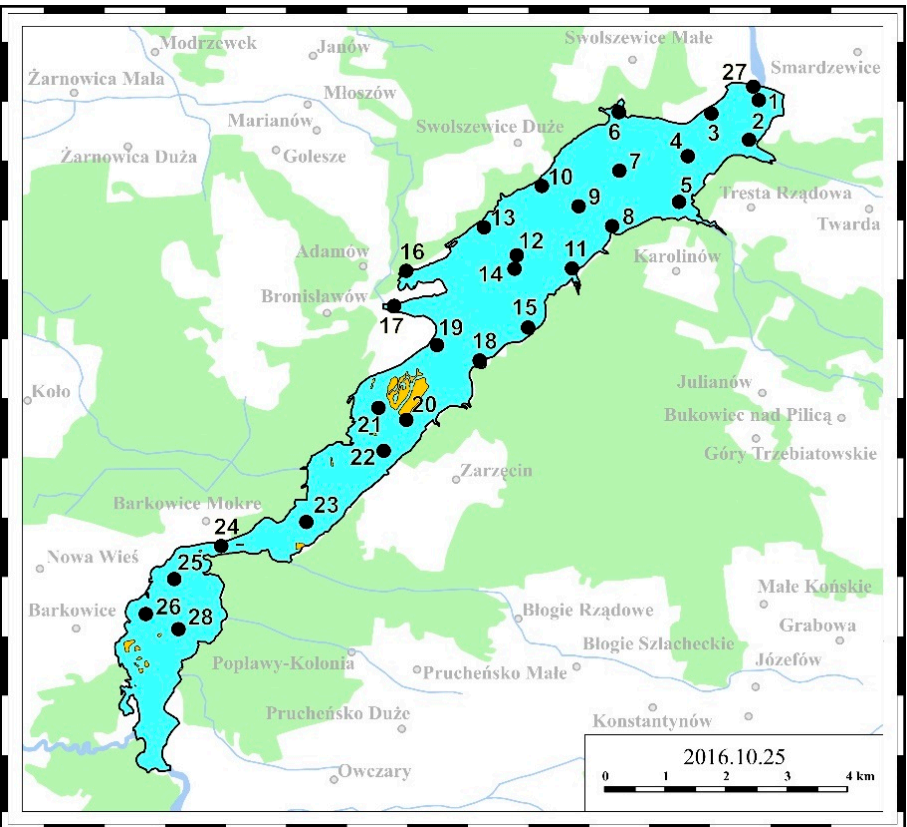
Location of water sampling points for laboratory tests.

The third part of the abovementioned measurement systems—the off-line measurement system—involved a laboratory analysis of water samples collected from the Sulejow Reservoir. About 30 water samples were collected at selected points of the Sulejow Reservoir along the route presented in Figure 3. Additionally, water samples were also collected from the Pilica River behind the dam, and the Luciaza River. An indicative location of the water sampling points is presented in Figure 4.

The following parameters characterizing water quality were identified in the collected samples: chemical oxygen demand (ISO 6060-1989), biochemical oxygen demand (EN 1899-1), total organic compound (DIN 38409-H3), nitrate nitrogen (PN-EN ISO 11905-1:2001) and phosphates (PN-EN ISO 6878-2006). During seasonal measurements carried out in May–October 2015 and April–November 2016 a total of 380 water samples were collected from the Sulejow Reservoir.

## 3. Results of Monitoring Studies

### 3.1. Stationary Monitoring

A stationary monitoring system activated within the project was based on a probe mounted on a buoy anchored in the reservoir. The system performed continuous measurements 24/7. In both measurement seasons 2015 and 2016 over 200,000 data items were collected, informing on seven parameters characterizing water quality in the northern part of the Sulejow Reservoir and data on meteorological conditions in the reference time.

#### 3.1.1. Results of Measurements of Meteorological Parameters

A meteorological module installed on a buoy anchored in the northern part of the Sulejow Reservoir was used to record the following meteorological data in the measurement periods: air temperature and humidity, wind speed and direction, atmospheric pressure and sunlight. The results of meteorological measurements are testimony to the fact that the period of air temperatures over 25 °C started relatively early in 2016—at the beginning of May—and then occurred again at the end of June 2016. In 2015 high temperatures were recorded in the whole of July (except for weekly periods with lower temperatures) and lasted until mid-August 2015. A general conclusion can be drawn that the profiles of air temperature changes in 2015 and 2016 were similar. The mean air temperature for the period between 20 May and 25 October was 16.4 °C in 2015, while in 2016 it was 16.9 °C. No major differences in the sunlight reaching the Sulejow Reservoir water were observed for both measurement seasons. The mean sunlight level between May and October in 2015 was 877 kWh/m^2^ and 871 kWh/m^2^ in 2016.

Significant differences were observed for atmospheric precipitation. In 2016 the number of hours with atmospheric precipitation amounted to 538, while the number of rainy hours in 2015 was only 274. In 2015, being a dry year, the climatic water balance for the Sulejow Reservoir area was negative.

#### 3.1.2. Results of Water Temperature Measurements

**Figure 5:**
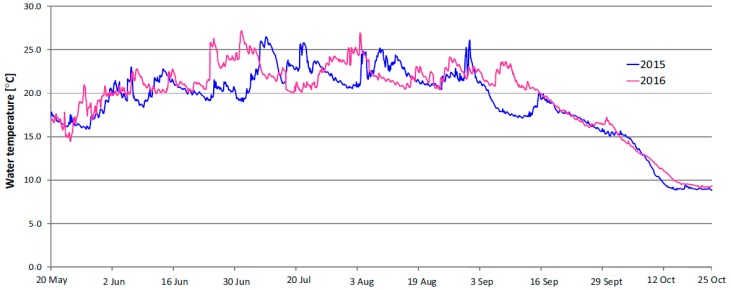
Profile of water temperature changes in the northern part of the Sulejow Reservoir between May–October 2015 and 2016.

Meteorological conditions which determined the weather in both measurement seasons affected the physicochemical parameters of water in the Sulejow Reservoir. The temperature of water in the reservoir was among the most essential parameters. The diagram presented in Figure 5 illustrates the evolution in the temperature changes in the northern part of the Sulejow Reservoir in the May–October period in 2015 and 2016.

In 2016 the water temperature reached 25 °C in mid-June but there was no day in July 2016 when the water temperature exceeded this value. Episodes with temperatures over 25 °C occurred at the beginning of August 2016. In 2015, elevated water temperatures in the northern part of the Sulejow Reservoir were recorded at the beginning of July and lasted (excepted for weekly periods with lower temperatures) to mid-August 2015. It should be highlighted that back in 2016, warm days with temperatures over 25 °C occurred also at the beginning of September. A general conclusion is that the overall profile of water temperature changes in the northern part of the Sulejow Reservoir was similar in 2015 and 2016. The mean water temperature between 20 May and 25 October was 19.0 °C in 2015, while in 2016 it was 19.6 °C. The number of hours with water temperature over 25 °C was 146 in 2015, while in 2016 it was 161. The maximum recorded water temperature in 2015 was 26.5 °C and 27.2 °C in 2016. Therefore statistically, the water in the northern part of the Sulejow Reservoir was slightly warmer in 2016 in relation to 2015.

#### 3.1.3. Results of Chlorophyll “a” Concentration Measurements

**Figure 6:**
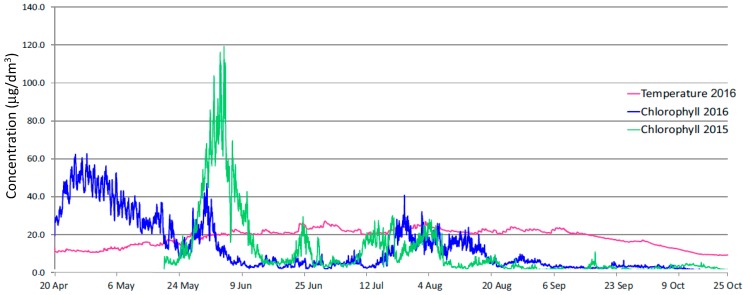
Profile of changes in chlorophyll concentration in the northern part of the Sulejow Reservoir in the period April–October in 2015 and 2016 (chlorophyll concentration in μg/dm^3^, water temperature in °C).

The EXO total algae PC sensor (chlorophyll and phycocyanin) was optimized for freshwater use. Figure 6 presents a profile of changes in chlorophyll “a” concentrations in the Sulejow Reservoir near the dam, in the measurement seasons 2015 and 2016.

In 2016, from the end of April to the beginning of May, despite low water temperatures (10–15 °C) chlorophyll concentrations ranged from 30 to 60 μg/dm^3^. A subsequent increase in the parameter concentration was observed in 2016, at the end of May. Unexpectedly, between 12 June and 12 July 2016, despite a significant increase in the water temperature, higher chlorophyll concentrations were not recorded. From mid-July to mid-August 2016, chlorophyll concentrations went up again but only to the level of 10–30 μg/dm^3^.

A comparison of the dynamics of chlorophyll concentration recorded in both seasons leads to the following conclusions: there is certain regularity in the occurrence of the maximum values of chlorophyll concentrations in the northern part of the reservoir—in 2015 the maximum increase was observed at the beginning of June. A similar increase was observed at the beginning of June 2016. Such a coincidence of changes was also recorded in the period from mid-June to mid-August 2015 and 2016. The similarities are well illustrated in the diagram in Figure 6 which was developed by overlapping data from two analysed years.

**Figure 7:**
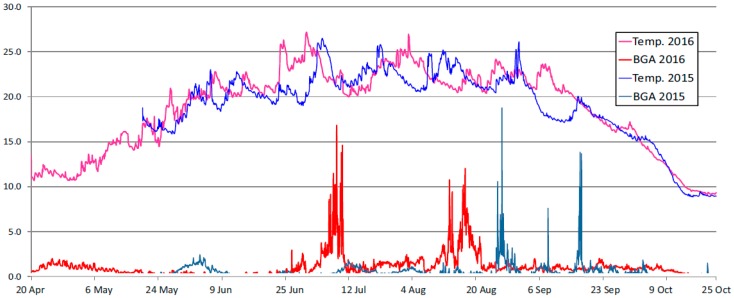
Diagram of changes in the water temperature and blue-green algae (BGA) concentration in the north part of the Sulejow Reservoir, in both analysed seasons (BGA concentration (μg/dm^3^); water temperature (°C).

First, chlorophyll concentrations in the northern part of the Reservoir, at the turn of June 2015 were extremely high as compared to concentrations in the reference period in 2016. Second, at the end of June 2015 the concentrations reached 20–30 μg/dm^3^, which were values not recorded in an analogical period in 2016. Figure 7 clearly shows a shift in the vegetation period of algae by about one week, at the turn of August in 2015 and 2016. Further studies and measurements carried out in subsequent years should be aimed at explaining the changes and confirming the findings of the early occurrence of high chlorophyll concentrations in the water, in the northern parts of the Sulejow Reservoir at the end of April and beginning of May.

#### 3.1.4. Results of Measurements of Blue-Green Algae (BGA)

Figure 7 presents a profile of changes in the concentration of blue-green algae (BGA) in the waters of the Sulejow Reservoir near the dam, in the measurement seasons 2015 and 2016.

The diagram presented in Figure 7 illustrates essential differences in the dynamics of blue-green algae blooming in the northern part of the Sulejow Reservoir in 2015 and 2016. High BGA concentrations between 5 and 15 μg/dm^3^ were observed twice in 2016: at the turn of August and in mid-August 2016. Such intensity of blooming was observed in 2015 much later—at the turn of September and in mid-September 2015. Further observations of the parameters should be aimed to explain the differences and identify the factors which cause significant differences in the dynamics of blue-green algae blooming in the waters of the Sulejow Reservoir.

#### 3.1.5. Results of Measurements of other Physicochemical Parameters of Water

Other parameters measured with the EXO2 probe, which characterized the quality of water in the Sulejow Reservoir, did not reveal such significant dynamic changes as water temperature and concentration of chlorophyll and BGA. Table 2 presents the results of measurements of other parameters of water near the dam, in the measurement periods 2015 and 2016.

The values of the parameters presented in Table 2 were calculated based on collections of data containing ca. 17,000 records registered by the EXO2 probe.

### 3.2. Results of Mobile Measurements

**Figure 8:**
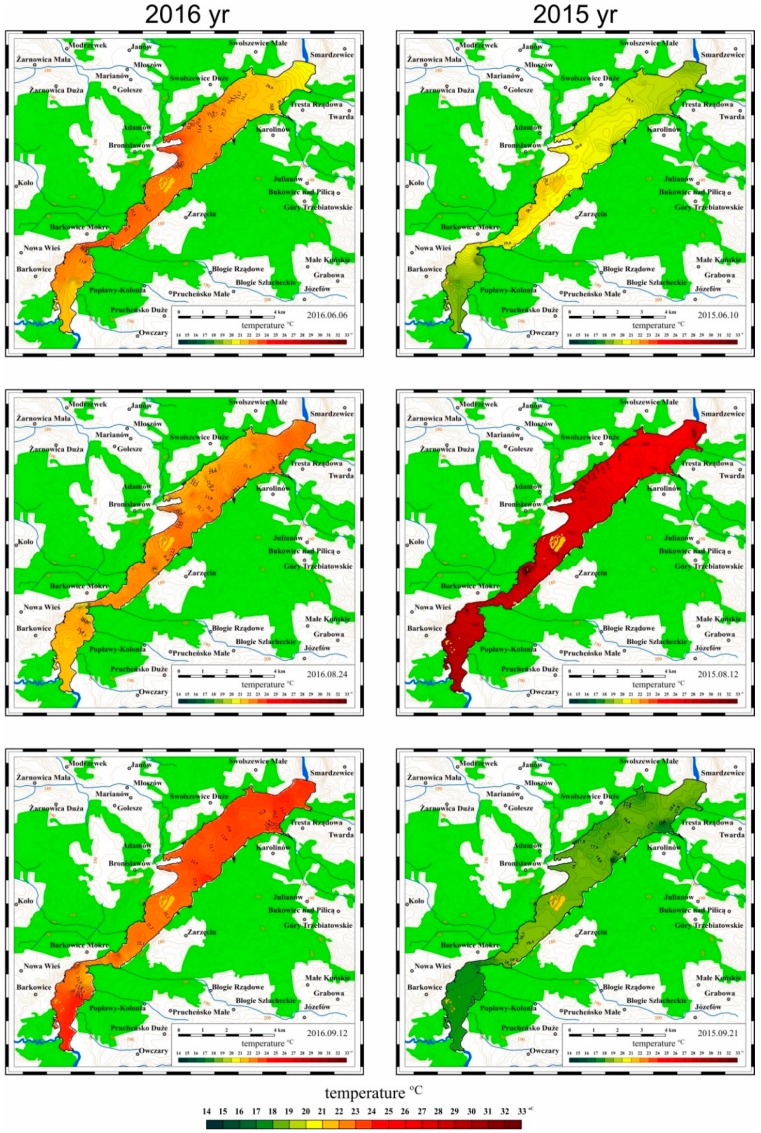
Spatial distribution of water temperature in the Sulejow Reservoir, between June and September 2015 and 2016 (yr: year).

**Figure 9:**
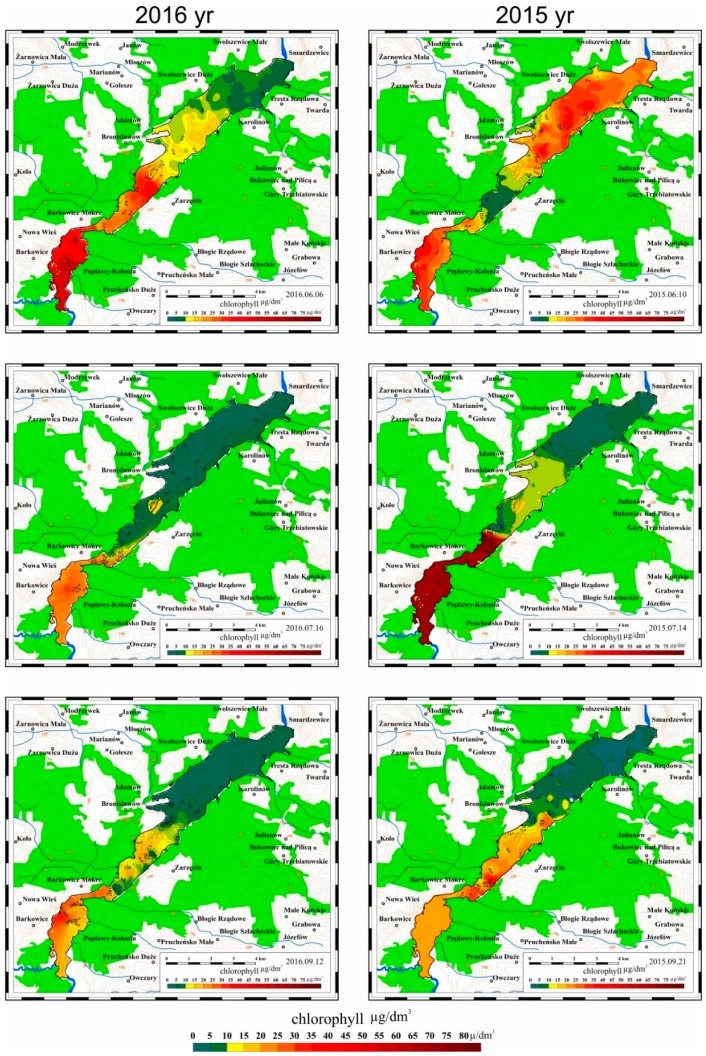
Spatial distribution of chlorophyll concentration in the Sulejow Reservoir between June and September 2015 and 2016.

**Figure 10:**
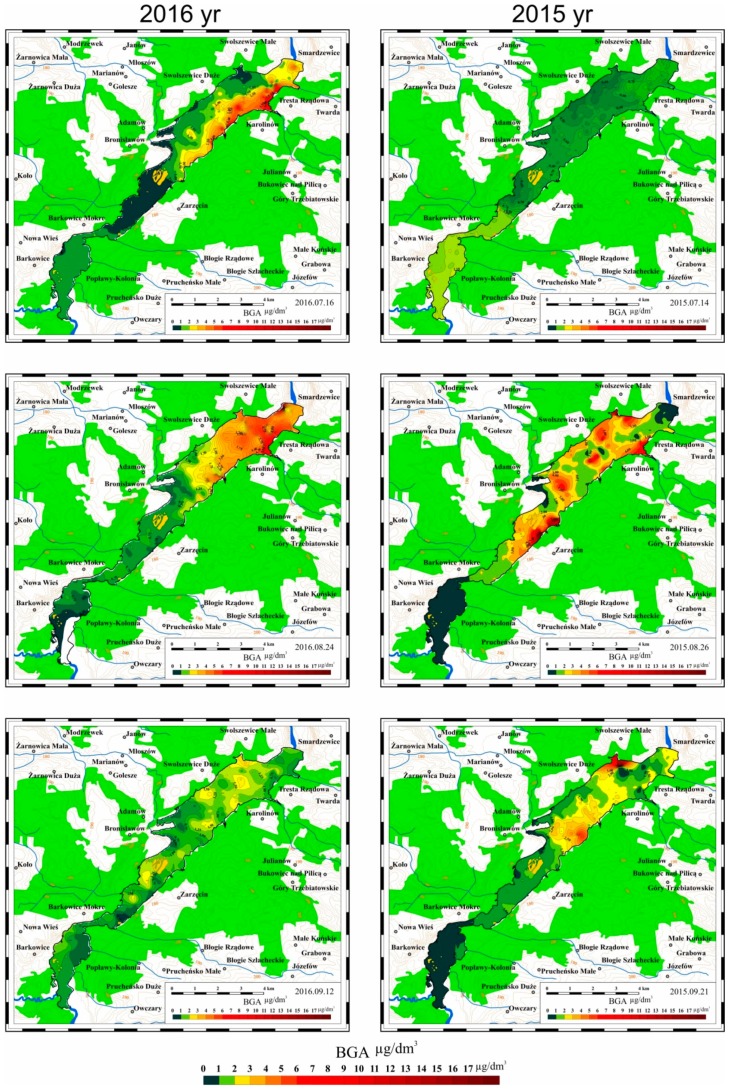
Spatial distribution of BGA (blue-green algae) concentration in the Sulejow Reservoir between June and September 2015 and 2016.

In 2015 and 2016 a mobile monitoring system was used for systematic measurements of selected parameters characterizing the quality of water in the Sulejow Reservoir. Based on the collected data, which were processed with ArcGIS software (Esri, Redlands, CA, USA), maps were developed illustrating the spatial distribution of the values of the measured parameters. The results of the examinations are presented in Figure 8, Figure 9 and Figure 10.

#### 3.2.1. Spatial Distribution of Water Temperature in the Sulejow Reservoir in 2015 and 2016

Changes in the water temperature in the Sulejow Reservoir fluctuate periodically but the spatial distribution of the changes i.e., the field of temperatures is fairly uniform. The temperature of water at a depth of up to 2 m in the whole reservoir is uniform and no places with particularly intensive water heating were observed.

The data presented in Figure 8 confirm the trend which was revealed in the analysis of the data from stationary measurements made in the northern part of the reservoir: in 2016 temperatures in the range of 25–27 °C were observed fairly early, in June and at the end of summer, i.e., in September 2016. The highest temperatures of water in 2015 (26–27 °C) occurred in August, while in mid-September 2015 the water temperature decreased quickly to 15–17 °C.

#### 3.2.2. Spatial Distribution of Chlorophyll Concentrations in 2015 and 2016

Elevated concentrations of chlorophyll in the waters of the Sulejow Reservoir were observed at the beginning of the vegetation period, i.e., at the end of May and beginning of June (see Figure 9).

An increase in chlorophyll concentration in the Sulejow Reservoir waters was poorly correlated with an increase in the water temperature, e.g., at the beginning of June 2015 the temperature of water in the reservoir ranged from 19–21 °C, while chlorophyll concentrations reached very high values between 60–90 μg/dm^3^. Similarly, at the beginning of June 2016, the temperature of water in the reservoir did not exceed 20–21 °C, while chlorophyll concentrations increased in the same period to 30–40 μg/dm^3^. In July, in both research periods, the temperatures of water in the reservoir were not high, while chlorophyll concentrations in the southern part of the reservoir were relatively high; especially in 2015 the concentration values were greater than 80 μg/dm^3^.

One should also note that high chlorophyll concentrations in the southern part of the Sulejow Reservoir persisted during the whole measurement period, i.e., from June to September, despite the fact that in the north-eastern part of the reservoir, chlorophyll concentrations decreased to 5–10 μg/dm^3^ from the beginning of July.

#### 3.2.3. Spatial Distribution of Blue-Green Algae (BGA) Concentration in the Sulejow Reservoir in 2015 and 2016

Blue-green algae occur naturally in freshwater ecosystems. Blooms can occur in response to favourable conditions, which include still or slow-flowing water, abundant sunlight, high temperatures and sufficient levels of nutrients. A feature characteristic of blue-green algae blooms is their non-uniform, focal nature, which is confirmed by the concentration distributions presented in Figure 10. Essential differences were also observed in the dynamics of changes in the BGA concentration in 2015 as compared to 2016. In 2016 intensive blooming of the blue-green algae was observed quite early—at the beginning of July and in mid-July. Most probably it could be attributed to an increase in the water temperature in the reservoir at the end of June and beginning of July 2016. In this period the water temperature in the Sulejow Reservoir ranged from 24 to 27 °C, and BGA concentrations reached values between 10 and 14 μg/dm^3^. Another episode of blooming took place in August and was also related to a rise in the water temperature which amounted to 24–25 °C from 4 August to 12 August 2016.

In 2015, high BGA concentrations (10–14 μg/dm^3^) were observed a bit later, in mid-July, and their intensity was also related to the increase in the water temperature in the reservoir, which reached values of 25–26 °C between 7 and 10 July. Moreover, in 2015 high BGA concentrations continued longer than in 2016, while at the beginning of September 2015 their concentration in some areas of the reservoir exceeded 10 μg/dm^3^. Most probably it was caused by an increase in the water temperature in the reservoir, which ranged between 24 and 26 °C from 31 August and 3 September 2015. An analysis of data concerning BGA concentration and dynamics of the parameter confirm the hypothesis about a significant impact of the water temperature on the occurrence of blue-green algae blooms in the area of the Sulejow Reservoir. Water in the south part of the reservoir, due to the low flow rate and formation of stagnant areas as well as lack of circulation of the water can cause the algae to over-compete against other organisms in the ecosystem and thus create massive algae blooms.

The data presented in Figure 10 suggest that high BGA concentrations occur in the north-eastern part of the Sulejow Reservoir. Blue-green algae blooms do not occur in one particular place of the reservoir but appear and disappear randomly, in the whole north-eastern area of the reservoir. Formation of areas with clusters of blooming algae is a result of the water flow hydrodynamics in the Sulejow Reservoir. The areas with elevated concentrations of BGA strongly correlate with the lines of current distribution plotted as a result of calculations and simulations of the water flow in the reservoir.

### 3.3. Results of Laboratory Tests

#### 3.3.1. Content of Phosphorus Compounds

**Figure 11:**
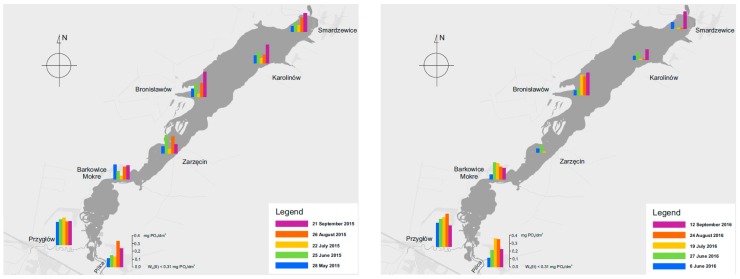
Changes in the concentration of phosphates in the Sulejow Reservoir in 2015 and 2016.

The content of phosphates in the surface waters of the Sulejow Reservoir ranged from 0.02 to 0.375 mg-PO_4_/dm^3^ (Figure 11). The highest concentrations were observed in the tributary rivers: the Pilica and Luciaza. In this case, the concentrations of phosphates varied between 0.10 to 0.48 mg-PO_4_/dm^3^.

The spatial distribution and dynamics of changes in the concentration of phosphates in the surface water of the Sulejow Reservoir show that higher concentrations are observed in the tributaries (Pilica and Luciaza). In the second half of September, in the north-eastern part of the reservoir, the concentrations of phosphates soared and the trend was observed in both measurement seasons. The studies also revealed a local increase in the concentration of phosphates in the reservoir waters, most probably caused by anthropogenic pressure. High concentrations in the post-vegetation period (September) can be caused by phosphates being released from the bottom and degrading phytoplankton.

#### 3.3.2. Content of Nitrate Nitrogen

**Figure 12:**
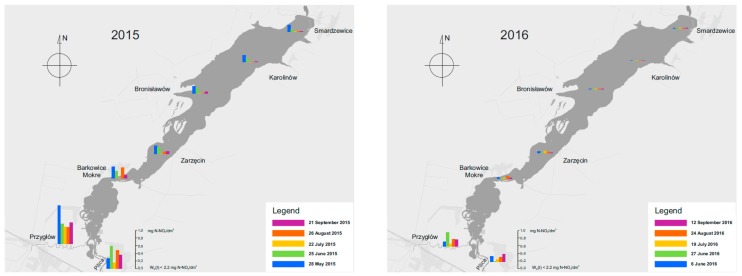
Changes in the concentration of nitrate nitrogen in the Sulejow Reservoir in 2015 and 2016.

The highest concentrations of nitrate nitrogen were observed in the Luciaza River in May 2015—0.98 N-NO_3_/dm^3^ (Figure 12). In other months the concentrations in the Pilica and Luciaza ranged between 0.1 and 0.76 mg N-NO_3_/dm^3^.

Much lower concentrations of nitrate nitrogen were measured though in the surface waters of the Sulejow Reservoir in comparison with the concentrations identified in the tributaries and ranged between 0.010–0.375 mg N-NO_3_/dm^3^ in 2015 and from 0.010 to 0.29 mg N-NO_3_/dm^3^ in 2016. The spatial distribution of nitrate concentrations in the reservoir waters and the dynamics of changes in time are testimony to the fact that surface run-offs from arable fields surrounding the Sulejow Reservoir are low, and the main stream of the compounds comes from the Pilica and Luciaza rivers. In a vegetation period, i.e., between July and August, the concentration of nitrogen ions in the water drops nearly to zero, which is evidence of systematic assimilation and/or denitrification by microorganisms in the water.

#### 3.3.3. Content of Total Organic Carbon, Chemical Oxygen Demand and Biochemical Oxygen Demand

**Figure 13:**
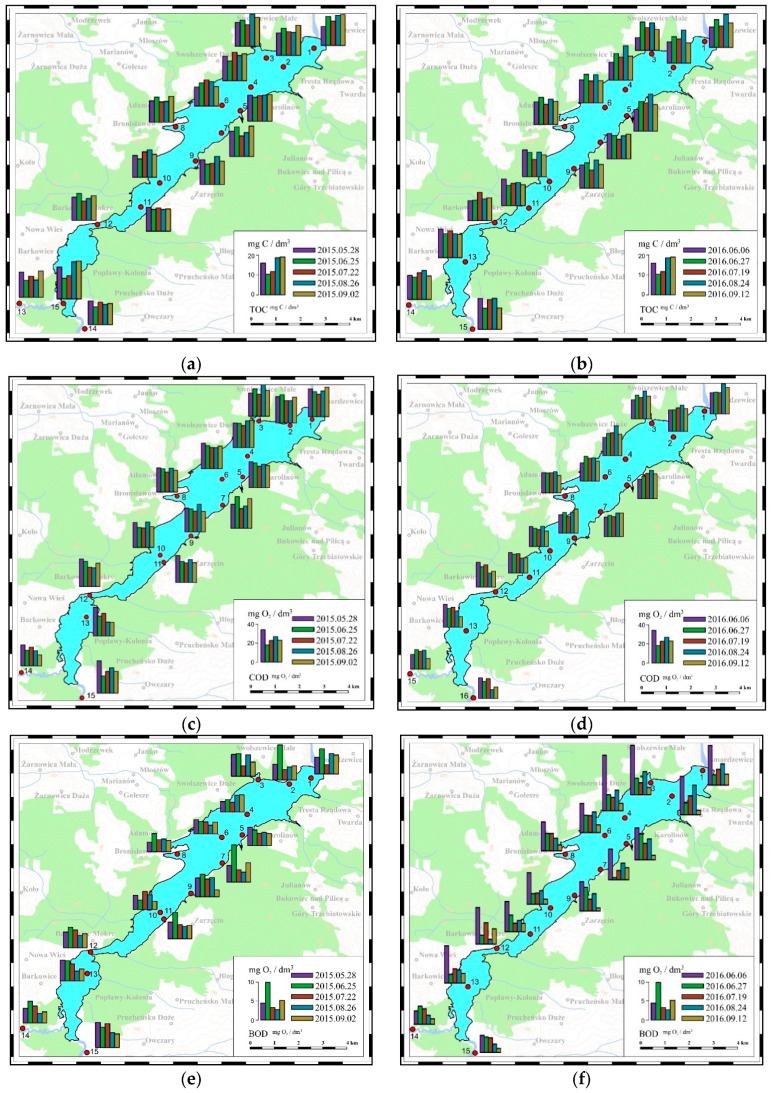
Changes in the concentration of TOC (Total Organic Carbon, (**a**,**b**)) (mg C/dm^3^), COD (Chemical Oxygen Demand, (**c**,**d**)) (mg O_2_/dm^3^) and BO D (Biochemical Oxygen Demand, (**e**,**f**)) (mg O_2_/dm^3^) in the Sulejow Reservoir in 2015 and 2016.

Concentration of TOC in the surface water of the Sulejow Reservoir is within the norm for I purity Class (15 mg C/dm^3^), only in a few analysed places does it fall in the second purity class (20 mg C/dm^3^). Similarly, the COD values are within the norm for I (25 mg O_2_/dm^3^) and second purity class (30 mg O_2_/dm^3^) and the concentrations in both monitoring seasons differ slightly, higher concentrations were found in the vicinity of the reservoir dam. The biggest differences are visible in BOD concentrations. Values often exceed standards for the 2nd purity class (6 mg O_2_/dm^3^). Higher concentrations were recorded in 2016 at the beginning of the growing season. The concentrations in the reservoir waters have higher values than in the supplying rivers, hence the conclusion that in the summer season the enrichment of the reservoir with organic substances takes place (Figure 13).

## 4. Conclusions

The water quality research carried out in the Sulejow Reservoir in the years 2015–2016 provided results showing the benefits of continuous monitoring in relation to periodic, traditional monitoring.

The data from monitoring measurements included in the ArcGis database were displayed in a graphic form and published as maps showing a temporary and spatial distribution of parameters characterizing the ecological status of the Sulejow Reservoir between May 2015 and April 2016.

For the first time, a multi-parameter probe was used to take mobile measurements, and the accuracy of the results was verified with reference to prior testing. Measurements during motorboat cruises provided a series of local data which were interpolated using the ArcMap and Spatial Analyst software and visualized as maps. The results were used to compare each of the measured water parameters in terms of spatial distribution and time period.

Analysis of the collected data allows the following conclusions to be formulated:

- profiles of air and water temperature changes in 2015 and 2016 were similar, significant differences were observed for atmospheric precipitation. Negative water balance caused limited surface runoff and decreased the concentrations of nutrient in reservoir waters.
- low nitrate and phosphate concentrations in the reservoir waters are not only caused by minor surface runoff in the analysed period, but also by a strong eutrophication and algal blooms. The spatial distribution and dynamics of changes in the concentration of biogenic substances show that higher concentrations were measured in the tributary rivers (Pilica and Luciaza).
- Blue-green algae occur mainly in the area of the high depths, lower flow rate and places of water stagnation, which favour the algae growth [14]. In contrast to the BGA, concentrations of chlorophyll “a” are higher in the shallower part of the reservoir.
- Concentrations of TOC, COD as a measure of organic matter content in waters, randomly exceed the standards for the 2nd class of water purity.
- Higher concentrations of BOD in the reservoir in comparison to the supplying rivers, recorded in 2016, were associated with active tourism in the summer season.
- Analysis of spatial distribution of biogenic substances in the reservoir shows significant differences in different parts of the lake, which means that single, stationary measurements do not deliver a realistic picture of the contaminants flow in the dam reservoirs.

Continuous monitoring provided a detailed view of the water quality parameters during the vegetation season enabling rapid detection of the values that exceeded the established norm and enabling implementation of the necessary practical steps to improve the water quality. As not all the parameters can be measured continuously laboratory tests are still required. Therefore, only the use of a complex monitoring system that combines different methods allows a complete picture of the ecological potential of the reservoir to be obtained.

## Figures and Tables

**Table 1:** Characterization of EXO measured parameters (https://www.ysi.com/EXO2) [11].

EXO Parameter Measured	Sensor	Range	Accuracy	Resolution
Ammonium (freshwater only)	Ammonium Sensor	0 to 200 mg/dm^3^ (0 to 30 °C)	± 10% of reading or 2 mg/dm^3^-N	0.01 mg/dm^3^
Blue-green Algae, Phycocyanin	Total Algae Sensor	0 to 100 μg/dm^3^; 0 to 100 RFU;	Linearity: R^2^ >0.999 for serial dilution of Rhodamine WT solution from 0 to 100 μg/cm^3^ BGA-PC equivalents	0.01 μg/dm^3^; 0.01 RFU
Chlorophyll	Total Algae Sensor	0 to 400 μg/dm^3^ Chl; 0 to 100 RFU	Linearity: R^2^ > 0.999 for serial dilution of Rhodamine WT solution from 0 to 400 μg/dm^3^ Chl a equivalents	0.01 μg/dm^3^ Chl; 0.01 RFU
Conductivity	Conductivity/Temperature Sensor	0 to 200 mS/cm	0 to 100: ±0.5% of reading or 0.001 mS/cm; 100 to 200: ± 1% of reading	0.0001 to 0.01 mS/cm (range dependent)
Depth—10 m	Integral, Non-vented Depth Sensor	0 to 10 m	± 0.04% FS (± 0.004 m)	0.001 m (auto-ranging)
Dissolved Oxygen, mg/dm^3^	Optical Dissolved Oxygen Sensor	0 to 50 mg/dm^3^	0 to 20 mg/dm^3^: ± 0.1 mg/dm^3^ or 1% of reading, 20 to 50 mg/dm^3^: ± 5% of reading	0.01 mg/dm^3^
pH	pH Sensor	0 to 14	± 0.1 pH units within ± 10 °C of calibration temp; ± 0.2 pH units for entire temp. range	0.01 units
Temperature	Conductivity/Temperature Sensor	−5 to 35 °C 35 to 50 °C	± 0.01 °C ± 0.05 °C	0.001 °C

RFU: relative fluorescence units, Rhodamine WT: Liguid form of dye, BGA-PC: blue-green algae-phycocyanin, Chl: chlorophyll, FS: accuracy as a percentage of full scale.

**Table 2:** Selected water quality parameters in the north part of the Sulejow Reservoir between April–October 2015 and 2016.

No.	Parameter Name	Unit	2015		2016	
			Mean Value	Standard Deviation	Mean Value	Standard Deviation
1.	pH value	-	8.39	0.26	8.79	0.31
2.	NH_4_^+^ concentration	mg/dm^3^	0.38	0.21	0.25	0.07
3.	Oxygen concentration	mg/dm^3^	9.23	2.43	10.75	3.09
4.	Conductivity	μS/cm	260.7	28.4	299.3	51.2
